# Research Progress of Self-Healing Polymer for Ultraviolet-Curing Three-Dimensional Printing

**DOI:** 10.3390/polym15244646

**Published:** 2023-12-08

**Authors:** Wenhao Liu, Zhe Sun, Hao Ren, Xiaomu Wen, Wei Wang, Tianfu Zhang, Lei Xiao, Guangpu Zhang

**Affiliations:** 1 National Special Superfine Powder Engineering Research Center of China, School of Chemistry and Chemical Engineering, Nanjing University of Science and Technology, Nanjing 210094, China; 13586987884@163.com (W.L.); sunzhe97529@163.com (Z.S.); rhmoon1112@163.com (H.R.); 15005161138@163.com (L.X.); 2 Science and Technology on Transient Impact Laboratory, No. 208 Research Institute of China Ordnance Industries, Beijing 102202, China; wenxm2908@163.com; 3 Science and Technology on Aerospace Chemical Power Laboratory, Hubei Institute of Aerospace Chemotechnology, 58 Qinghe Road, Xiangyang 441003, China; wang7469061@163.com (W.W.); charlestfzhang@sina.com (T.Z.)

**Keywords:** self-healing, UV-curing, 3D printing, polymer

## Abstract

Ultraviolet (UV)-curing technology as a photopolymerization technology has received widespread attention due to its advantages of high efficiency, wide adaptability, and environmental friendliness. Ultraviolet-based 3D printing technology has been widely used in the printing of thermosetting materials, but the permanent covalent cross-linked networks of thermosetting materials which are used in this method make it hard to recover the damage caused by the printing process through reprocessing, which reduces the service life of the material. Therefore, introducing dynamic bonds into UV-curable polymer materials might be a brilliant choice which can enable the material to conduct self-healing, and thus meet the needs of practical applications. The present review first introduces photosensitive resins utilizing dynamic bonds, followed by a summary of various types of dynamic bonds approaches. We also analyze the advantages/disadvantages of diverse UV-curable self-healing polymers with different polymeric structures, and outline future development trends in this field.

## 1. Introduction

**Figure 1:**
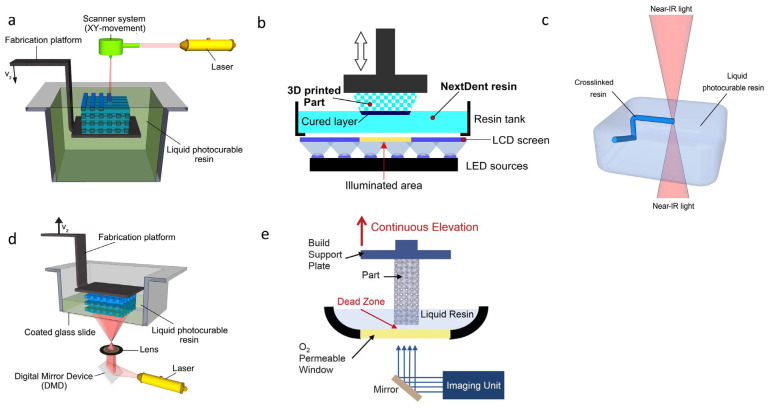
(**a**) Schematic diagram of stereolithography (SLA) [42]. (**b**) Schematic diagram of liquid crystal display (LCD). Reprinted with permission from reference [43], 2021, ELSEVIER. (**c**) Schematic diagram of two-photon 3D printing (TPP) [42]. (**d**) Schematic diagram of digital light processing (DLP). Reprinted with permission from reference [42], 2012, ELSEVIER. (**e**) Schematic diagram of continuous liquid interface production (CLIP). Reprinted with permission from reference [44], 2015, SCIENCE.

3D printing technology has been widely used since the 1980s in many fields, such as automobile manufacturing [1], the aerospace industry [2] and biomedicine [3]. The high RTM (resolution/time for manufacturing ratio) of 3D printing technologies which use ultraviolet light as a curing source makes it popular for practical applications [4]. Molecules containing light absorption units, namely chromophores (such as C=C, C=O), are in an excited state after being irradiated by a certain wavelength of ultraviolet light. The excited-state molecules undergo a crosslinking reaction and change from the original liquid state to the solid state. The principle of UV-curing 3D printing technology is to use ultraviolet light to selectively cure the photosensitive resin under the control of digital signals. The liquid resin is attached to the previous curing layer and then cured. The process is repeated until a complete printed product is formed [5]. UV-curing molding technologies include stereolithography (SLA) [6], digital light processing (DLP) [7], liquid crystal display (LCD) [8], and so on. A working principle diagram of several photocurable 3D printing technologies is shown in Figure 1, and their advantages and disadvantages are compared in Table 1. The stable cross-linking networks make prepared products difficult to recycle and reprocess when they generate microcracks and defects. At the same time, volumetric shrinkage is unavoidably generated as the polymerization reaction proceeds, and the van der Waals forces between the monomers are converted into covalent bonds, resulting in a shorter distance between the monomers [9,10]. Based on these factors stated above, the accuracy and mechanical properties of the prepared products are reduced. Therefore, some researchers have introduced dynamic bonds into polymer chains to realize the self-healing function of the materials. The dynamic “breaking-recovering” reversible process of bonds helps to release intramolecular shrinkage stress produced during the polymerization process, thereby reducing the volume shrinkage [11,12].

Self-healing materials can repair their defects and restore their original performances under certain external stimuli (light or heat). Up until now, a variety of self-healing materials have been successfully prepared and used, such as concrete [13,14], metallic [15,16], and polymer materials [17,18]. Self-healing polymers are widely used in biomedicine [19,20], protective coatings [21,22], and other fields [23]. Based on the different self-healing mechanisms, such polymers are divided into extrinsic [24] and intrinsic types [25]. Extrinsic self-healing polymers are prepared by filling the raw material resin with micro-/nano-structures such as micro-capsules [26] and micro-fibers [27] containing healing agents. For instance, Shinde [28] and Sanders [29] et al. prepared a UV-curable resin containing self-healing micro-capsules, achieving good 3D printability and a satisfactory self-healing effect. However, the number of repairs was limited because the healing agent was exhausted when the crack increased [30,31]. In contrast, the healing process in intrinsically self-healing polymers is based on the fracture and recombination of dynamic bonds in the polymer chains. Dynamic exchange reactions are activated by external stimuli and can theoretically be exchanged infinite times. Therefore, intrinsic self-healing polymers have attracted a great deal of attention. Dynamic bonds are divided into dynamic non-covalent bonds (including ionic bonds [32], hydrogen bonds [33], coordination bonds [34], etc.) and dynamic covalent bonds (including Diels–Alder bonds [35,36], ester exchange bonds [37], disulfide bonds [38], imine bonds [39], etc.). Compared with dynamic non-covalent bonds, dynamic covalent bonds usually have higher bond energy [40] and endow materials with better mechanical properties. Thus, to realize self-healing, a high temperature or energy is necessary [41], which causes some restrictions on the application of dynamic covalent bonds.

In summary, it is highly desirable to develop photosensitive 3D printing resins with a self-healing ability. At present, UV-curable dynamic-bond polymers have been intensively investigated, and studies aim to introduce a self-healing function into UV-curing 3D printing products. Many dynamic bonds, including disulfide bonds, ester exchange bonds, Diels–Alder bonds, hydrogen bonds, ionic bonds, and metal coordination bonds, have been used to construct UV-curable 3D printing materials. This paper mainly introduces the common dynamic bonds in UV-curing 3D printing polymers and evaluates the performance advantages and applications of different materials.

**Table 1:** Comparison of various popular UV-curing 3D printing technologies [45,46].

Name	Operation Principle	UV-Curing Mechanism	Advantage	Disadvantage	Application
SLA	Laser beam single-point printing	Free radical and hybrid curing	Mature technology, form large size device	Slow curing speed	Dentistry, mold, automobile
DLP	Projection printing	Free radical curing	Fast curing rate, high precision	Form small size device	Medical care, jewelry, education
CLIP	Projective continuous printing	Free radical and thermocuring	Extremely fast curing speed	Expensive resin and equipment	Sports, cars
MJP ^1^	Multi-nozzle printing	Free radical and hybrid curing	High precision, colorfulness	Expensive equipment	Consumer goods, medical care jewelry
TPP	Dual Laser Beam Printing	Free radical curing	Extremely high precision	Expensive equipment, complex process	Microelectronics, art, scientific research
LCD	Liquid crystal imaging printing	Free radical curing	Fast curing speed, low cost	Short service life	Jewelry, mold manufacturing

^1^ MJP [47] technique is also called PolyJet.

## 2. Dynamic Covalent Self-Healing Polymers for UV-Curing 3D Printing

Due to the different types and properties of dynamic bonds, polymers with different properties can be constructed. Under certain external stimuli, these polymers achieve restoration by rearranging their dynamic covalent bonds. In general, dynamic covalent bonds exhibit high bond energy and stability, which endow the polymer with a higher mechanical strength and modulus.

### 2.1. Dynamic Disulfide-Bond

**Figure 2:**
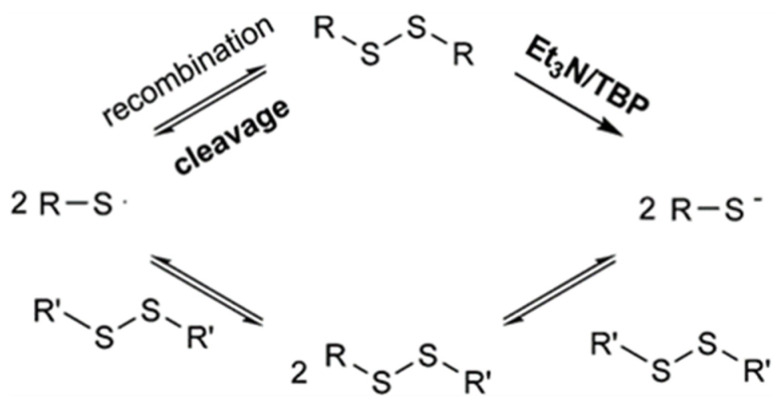
Disulfide exchange through the formation of sulfur-based radicals and sulfur-based anions. Reprinted with permission from reference [50], 2016, ELSEVIER.

The relatively low bond energy of the disulfide bond (about 250 kJ·mol^−1^) [48] makes it easily activated at room temperature [49] or by a certain intensity of UV-light irradiation [38]. Under the action of free radicals or anionic intermediates, disulfide bonds are activated and then recombined to achieve self-healing. The disulfide bond exchange reaction is shown in Figure 2.

Li et al. [51] reported a UV-curable polyurethane acrylate resin (PUSA) based on disulfide-bond self-healing for DLP 3D printing. The authors first reacted bis(2-hydroxyethyl) disulfide and polyethylene glycol with isophorone diisocyanate to prepare an isocyanate-terminated prepolymer (PUSA). Then, the isocyanate group was terminated with 2-hydroxyethyl acrylate. The prepared PUSA showed a mechanical self-healing performance under UV irradiation. The tensile strength and elongation at break were 3.39 ± 0.09 MPa and 400.38 ± 14.26%, respectively. After healing at 80 °C for 12 h, the spline could withstand large tensile deformation, and the healing efficiency reached 60% of the initial value. Even after healing for the third time, the tensile strength of the samples reached 2.05 ± 0.34 MPa.

The formula for self-healing efficiency is as follows:

(1) $$\eta =\frac{\sigma_{healed}}{\sigma_{original}}$$

In the formula: *η*(*η_stress_*/*η_strain_*)—tensile strength (stress/strain) repair efficiency; *σ_healed_*—tensile strength after repair; *σ_original_*—original tensile strength. A series of control experiments confirmed the decisive effect of the disulfide bond on the self-healing. The prepared UV-sensitive resin exhibits moderate viscosity, good fluidity, and a fast UV-curing rate. The double-bond conversion rate after 60 s of UV irradiation is about 80%. Moreover, it shows good compatibility with commercial DLP 3D printers and can be used to print various 3D products. The printed products have high definition, smooth surfaces, and preserved mechanical and self-healing functions. Owing to its excellent performance, such a polymer has great application potential in the fields of UV-curable coatings, adhesives, and inks.

**Figure 3:**
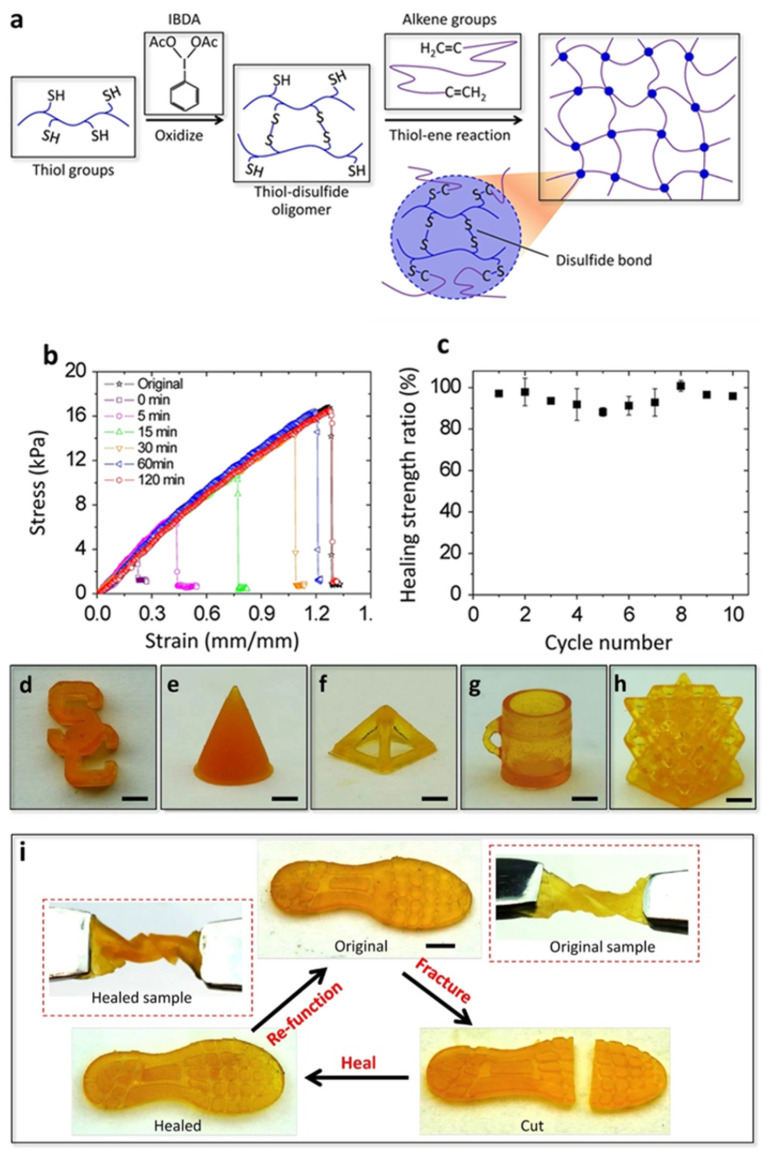
(**a**) Molecular design of the self-healing elastomer. (**b**) Nominal stress–strain curves of the original and self-healed experimental elastomers for various healing times. (**c**) Healing strength ratios of the experimental elastomers for 10-cycle healing tests (each 2 h at 60 °C) (**d**–**h**). The manufactured samples. (**i**) Self-healing of a shoe pad sample (scale bars: 4 mm). Reprinted with permission from reference [53], 2019, Springer Nature.

Thiolene photopolymerization is a much more efficient way to crosslink elastomers than the copolymerization of double bonds with acrylate monomers [52]. So Yu et al. [53] reported a UV-printable self-healing elastomer based on sulfhydryl and disulfide bonds. Under UV-light irradiation, the polymerization of sulfhydryl and alkene [54,55] activated rapidly. The metathesis reaction of disulfide bonds in the system endows the elastomers with a self-healing function. It is worth noting that the number of sulfhydryl groups affects the UV-curing rate, while the number of disulfide bonds affects the healing performance. This research on the interaction between the UV-curing speed and healing efficiency paved the way for a subsequent similar system [56], in which a balance between two parameters was achieved. In addition, the ratio of the cross-linking groups to the self-healing groups can be regulated via the oxidation reaction of the thiol group, which, in principle, facilitates the preparation of polymers with the desired mechanical performance and self-healing function. Thiolated (mercaptopropyl) methylsiloxane-dimethylsiloxane (MMDS) was oxidized by Iodobenzene diacetate (IBDA) [57] to form oligomers containing both thiol and disulfide functional groups (Figure 3a). Then, the oligomers were reacted with vinyl-terminated polydimethylsiloxanes (V-PDMS) with a double bond to form a solid elastomer. V-PDMS has a relatively low viscosity (below 200 cSt), which is suitable for the stereolithography process. The printed elastomers have a high resolution (up to 13.5 μm) and excellent self-healing performance. After healing at 60 °C for 2 h, the apparent cracks reconnected and the tensile strength was restored (Figure 3b). High healing performance is still achieved even after more than 10 cycles of self-healing tests (Figure 3c); however, the Young’s modulus of the experimental elastomer is relatively low (17.4 kPa). The research on the theoretical model of self-healing guides the understanding of self-healing polymers and the use of dynamic bonds [58,59,60].

Zhang et al. [38] prepared a chemically cross-linked ionic gel (ionogel) system with the synergistic effect of dynamic disulfide bonds, hydrogen bonds, and ion-dipole interaction. The synergistic effect of various forces is beneficial to enhancing the material’s performance. Polyurethane acrylate with disulfide bonds was synthesized and then compounded with the diluent monomer 4-Acryloylmorpholine, the ionic liquid, and a photoinitiator to obtain a UV-sensitive resin. The UV-cured elastomer shows good transparency and can be modified by adding different color additives. By adjusting the mass ratio of the raw materials, the tensile strength of the ionogels can reach 7.42 MPa, and the elongation at break is 977%. The mechanical performances of the ionogels remained after continuous cyclic tensile/compression tests. The micro-pressure sensors were prepared by DLP3D printing technology. The sensors have excellent elasticity and durability, and good response sensitivity and self-healing functions. The healing efficiency after UV irradiation for 10 min is greater than 99%. The results confirm that the ionogel has great application prospects in wearable ionotronics and intelligent soft robotics.

Gomez et al. [61] presented a UV-curable, self-healing elastomer system that can achieve ultra-high elongation (1000%). The elastomer realized the modular manufacturing of soft robots by DLP printing technology. The acrylic elastomers have chain and reticular structures that can be adjusted by adding small amounts of dithiol and dynamic thioether crosslinkers. Through a thermally induced reverse Michael reaction, the free thiol and acrylate portions rapidly combine to form new thioether bonds for effective polymer diffusion and self-healing. The polymer has a complete self-healing ability in multiple damage/repair cycles via dynamic disulfide-bond exchange. These elastomers are compatible with self-healing stretchable electronic devices. It was used for 3D printing and integrated to form highly complex, large-scale functional soft robots. The presence of dynamic bonds can eliminate the need for adhesives or complex connectors during the connection of the sub-components. This is of practical significance for simplifying the component process.

### 2.2. Transesterified

**Figure 4:**
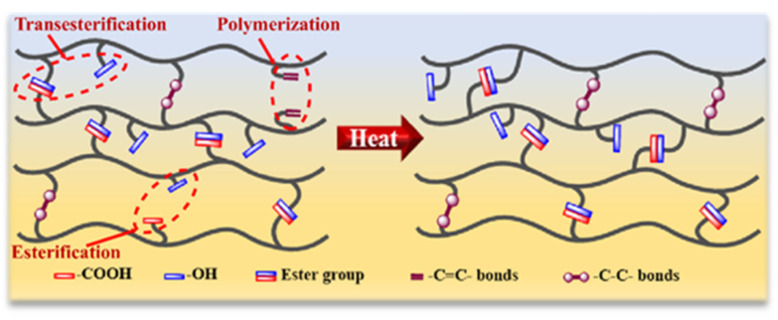
Illustration of the repair mechanism based on the transesterification. Reprinted with permission from reference [64], 2021, ELSEVIER.

Ester bonds are widely found in a variety of commercial thermosetting polymers, such as epoxy resins, unsaturated polyester resins [62], alkyd resins [63], etc. As shown in Figure 4, the hydroxyl and ester groups contained in the polymers can undergo a rapid dynamic transesterification reaction [64] at high temperatures, which does not change the topology of the polymer, and provides it with good self-healing performance.

Grauzeliene et al. [37] used 2-Hydroxy-2-Phenoxypropyl acrylate and bio-based acrylate epoxidized soybean oil to form a new type of vitrimer that can be DLP printed. The rich hydroxyl and ester groups in the system provide the necessary conditions for the transesterification reaction. The printed product has a two-way shape memory, 47% self-healing performance, 31% re-processability, and 100% alcohol degradation recovery performance. This product was used as artificial muscles and actuators; however, its high healing temperature (200 °C) and re-processing temperature (180 °C) limit its applications. The same authors designed and synthesized a vitrimer based on glycerol and vanillin for environmental protection strategies [65]. Owing to the high rigidity and thermal stability of the main chain of vanillin, the vitrimer exhibits excellent performance. Acrylated epoxidized soybean oil was first used in UV-curable resins for the synthesis of vitrimer. The use of environmentally friendly soybean oil as a raw material is in line with today’s green development concepts.

### 2.3. Imine Bond

The dynamic exchange reaction of imine bonds is also used in the field of self-healing [66,67]. The thermally initiated imine exchange reaction is mild and occurs rapidly without considerable side reactions. The imine bonds can recombine at room temperature [68].

**Figure 5:**
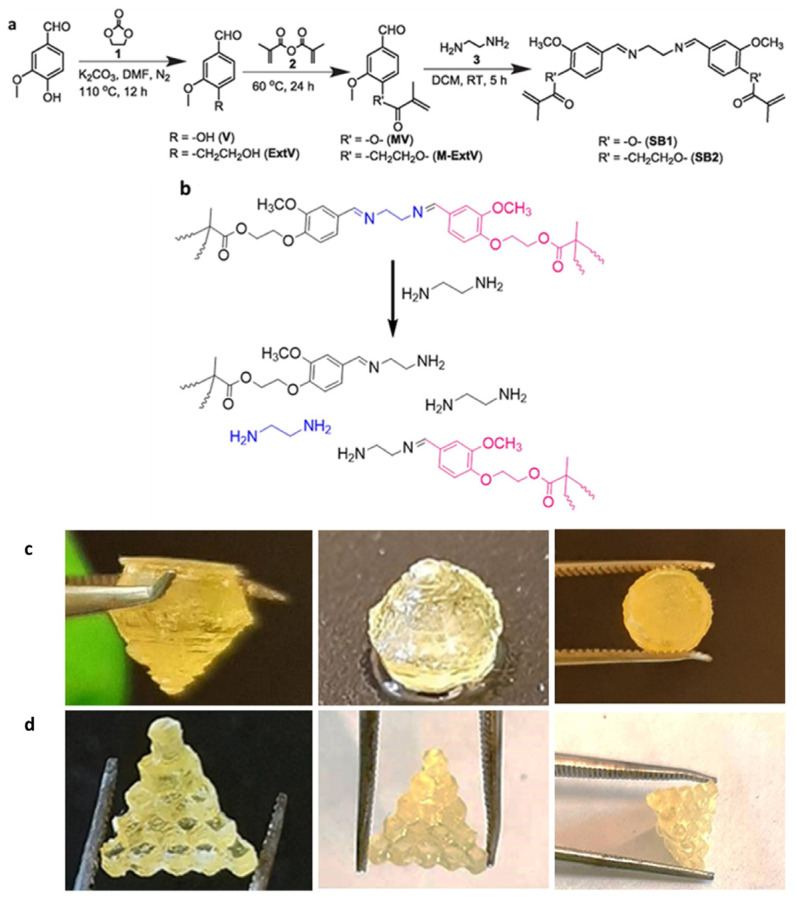
(**a**) Scheme of reactions for the synthesis of resins. (**b**) Suggested transamination pathway leading to the solubilization of resin in ethylenediamine. (**c**) 3D-printed diamond; (**d**) 3D-printed stairs. Reprinted with permission from reference [69], 2021, ELSEVIER.

Liguori et al. [69] prepared a vanillin-based UV-curable resin with self-healing ability that can be used as a DLP printing material and can be mechanically and chemically recovered by heat treatment. The Schiff base reaction between the aldehyde functional group of vanillin and the amino group of ethylenediamine generates an imine functional group. The hydroxyl functional group of vanillin reacts with acrylic anhydride to obtain a UV-reactive double bond (Figure 5a). As a result, the cured thermosetting material exhibits a high fracture strength (17.3 ± 3.9 MPa), extensibility, self-healing, and thermal re-processability due to the presence of a cross-linking structure and imine bonds. In addition, it can be chemically recovered via transamination in ethylenediamine (Figure 5b), and the obtained oligomeric product with amine end groups can be used to produce novel thermosetting films. Tensile tests showed that the elastic moduli of mechanically and chemically recycled thermosetting plastics are similar, whereas the elastic moduli of self-healing samples are slightly higher and the elongation and fracture stress are slightly lower. This resin was demonstrated to be suitable for producing 3D objects via DLP printing (Figure 5c,d), although the printing accuracy is not as good as that obtained using non-biological resins [70].

To explore the effect of different Jeffamines on the properties of cured products, Cortes-Guzman et al. [39] adjusted the type and chain length of the polyamines, and five thermosetting polymers with different mechanical performances were obtained. The Young’s moduli of these range between 2.05 and 332 MPa. They were applied to various applications based on their different performances. Since triamine provided a higher cross-linking density network than the diamine, the former was found to show a higher ultimate tensile strength (UTS) and Young’s modulus; however, the degree of freedom of the polymer chains decreased and the elasticity was inferior to that of the diamine system. Because of the simple synthesis process and commercial availability of Jeffamines, this series of resins provides a promising alternative to commonly used printing formulations. Furthermore, such bio-based polymers are expected to replace those made from nonrenewable resources [71].

### 2.4. Diels–Alder Bond

The reversible Diels–Alder addition reaction occurs between electron-deficient diolefins and electron-rich conjugated dienes at low temperatures and the reverse reaction is activated at high temperatures [72]. It is widely used in the field of self-healing polymers. A representative example is the reaction between furan and maleimide [73,74]. Structures containing a Diels–Alder bond network exhibit higher intermolecular force and better mechanical performances than non-covalent bonds [75,76]. Until now, the reversible Diels–Alder reaction has been scarcely applied in the field of 3D printing, because it requires a high temperature to initiate healing.

**Figure 6:**
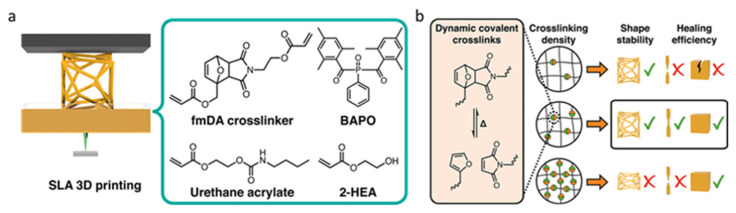
(**a**) Formulation of self-healing resins. (**b**) By varying the concentration of dynamic cross-links in resins, it is possible to balance the self-healing efficiency and the shape stability of printed objects. Reprinted with permission from reference [35], 2021, American Chemical Society.

Durand-Silva et al. [35] studied the effect of thermally reversible Diels–Alder cross-linking agents on the shape stability and self-healing performances of UV-printable resins. Aiming to optimize the ratio of raw materials to provide a self-healing function (>99%) without affecting the shape stability of the printed products, the authors prepared a UV-sensitive self-healing resin using acrylic furan−maleimide (fm) cross-linking agent and 2-Hydroxyethyl acrylate (2-HEA) as a reactive diluent (Figure 6a).

The results showed that the extent of the effect on the 3D printing shape increased with the concentration of the dynamic covalent cross-linking agent (Figure 6b). The healing efficiency did not show a linear relationship with the concentration of the covalent cross-linking agent, and no self-healing phenomenon was observed even at a high concentration, in contrast to general beliefs. Similar to other self-healing printing polymers, reliable self-healing polymers based on furan−maleimide Diels–Alder (fmDA) adducts must strike a balance between shape stability, thermal stability, and dynamics.

## 3. Dynamic Non-Covalent Self-Healing Polymers for UV-Curing 3D Printing

The activation of dynamic bonds usually requires conditions such as a high temperature or energy input [77], which involves additional energy use. The bond energy of dynamic non-covalent bonds is often lower than that of covalent bonds. The polymers formed through dynamic non-covalent bonds are less susceptible to the external environment and, therefore, require lower-intensity stimulation to heal the damage [78].

### 3.1. Hydrogen Bond

Hydrogen bonding is a common physical interaction that is much weaker than covalent bonds. When hydrogen atoms are adjacent to highly electron-rich atoms such as N, O, or F, reversible cross-linking networks are established via electrostatic interactions in polymer chains. Increasing the temperature can weaken the hydrogen bonding force, which is conducive to the movement of polymer chain segments. When the temperature decreases, the hydrogen bond will be regenerated again [79]. The hydrogen bond is suitable for constructing intrinsically self-healing materials and, thus, merits considerable research attention.

**Figure 7:**
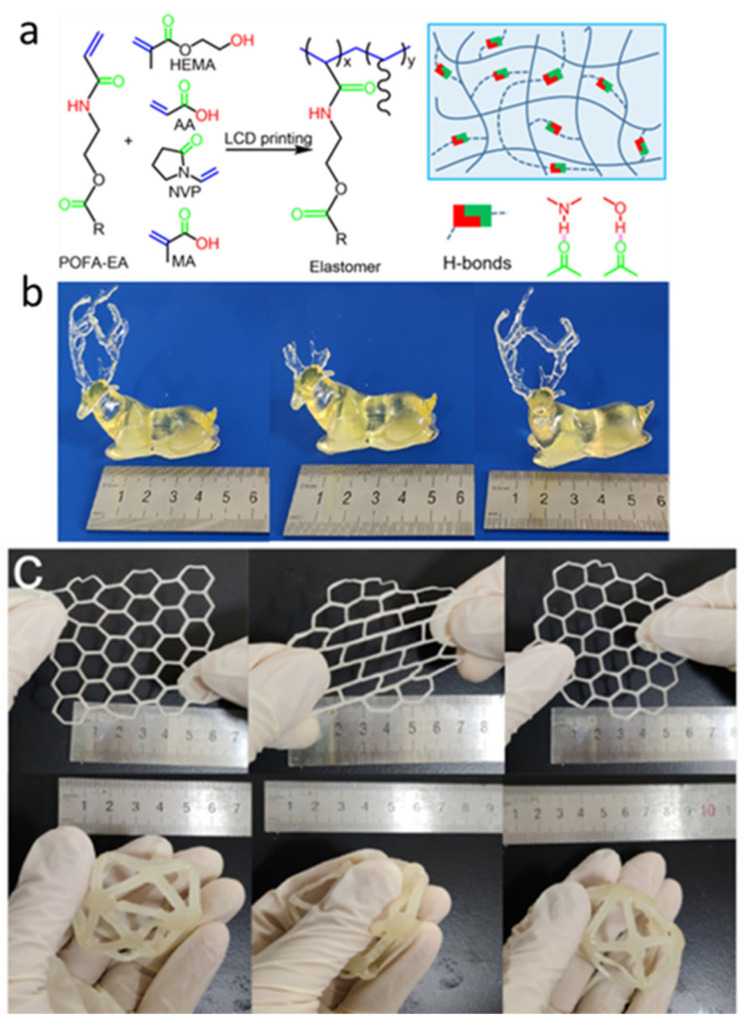
(**a**) Reaction mechanism of PO-based elastomers formed using POFA–EA and comonomers (HEMA, AA, NVP, or MA). (**b**) Photographs demonstrating self-healing in 3D deer model. (**c**) Reversible stretching and deformation of 3D objects. Reprinted with permission from reference [80], 2021, American Chemical Society.

Wu et al. [80,81] selected a series of UV-curable methacrylic acid and acrylic monomers as inks to apply in UV 3D printing. A vinylated palm oil monomer (POFA-EA) was selected as the raw material for the printing of thermoplastic polymers. The ink was obtained by blending N-vinyl-2-pyrrolidone (NVP) and acrylic acid (AA) (Figure 7a), and various bio-based thermoplastics were successfully printed using LCD technology. The ink exhibits a high double-bond conversion rate of 73–85% within 8 s, which meets the polymerization requirements for LCD printing. The amide structure in the vinylated palm oil monomer forms hydrogen bonds with polar monomers, including NVP and AA, enable the transparent printed products to exhibit high stretchability (Figure 7c) and self-healing abilities (Figure 7b), as well as easy processing and recycling abilities. The authors also explored the printability of a series of UV-curable monomers such as N-hydroxyethyl acrylamide (HEAA), AA, NVP, etc. The formation of strong hydrogen bonds between the –NH and C=O groups of polymer chains allows the polymers to meet the LCD requirements. Even mixing with other monomers does not affect the printability of N-hydroxyethyl acrylamide. This study guided the selection of the appropriate printing monomers. The viscous elastomer prepared in this paper can adhere to human skin, enabling repeated stripping–adhesion after 10 cycles without leaving any residue or causing skin irritation. These excellent features were exploited to prepare biosensors and wearable devices, demonstrating the great potential of this elastomer as a biomedical material.

Using natural tannic acid, choline chloride, and hydroxyethyl methacrylate as raw materials, Zhu et al. [33] developed novel ternary polymerizable deep eutectic solvents (PDESs) using the one-pot method. Density functional theory analysis indicated that hydrogen bonds and van der Waals interactions were the main driving forces for the formation of PDESs. The prepared PDESs showed high bio-based content (40.13–52.96%) and excellent mechanical performances (tensile stress, 5.45–13.71 MPa; toughness, 13.40–23.29 MJ·m^−3^). The degree of healing reached 85.2–88.5% after heating at 80 °C for 24 h, and the performances after healing were still superior to most reported PDES materials [82,83,84]. Because tannic acid exhibits natural UV-light absorption [85] and anti-bacterial properties, these new PDESs can be directly used for LCD printing without additional inhibitors [86]. The penetration depth of the PDES sample was low (0.192 mm), and the different structures that were printed all showed high resolutions. The penetration depth and critical exposure energy [87] of UV-sensitive resin were investigated, which paved the way for the further improvement of printing accuracy. Overall, the developed tannic acid-based PDESs can serve as ideal materials for high-resolution 3D printing, and are equipped with both green and multi-functional properties.

**Figure 8:**
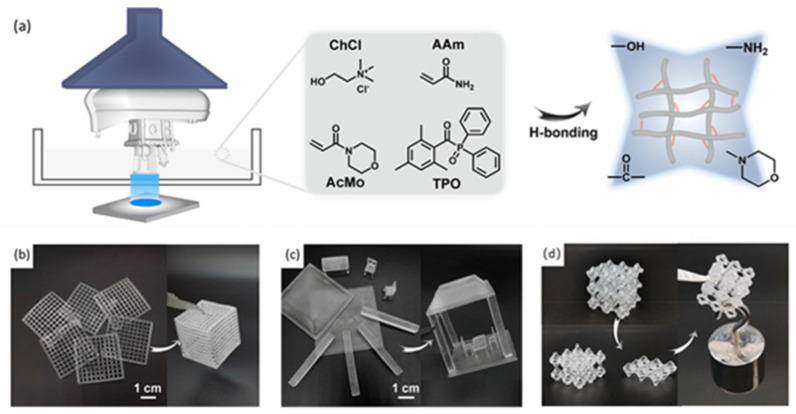
(**a**) LCD printer for the fabrication of 3D objects, the composition of the resin, and the hydrogen-bonding network in the resin. (**b**) 2D gridding to a 3D-box structure; (**c**) small parts to a complex pavilion-like building. (**d**) Photographs showing the self-healing capability of a 3D-printed lattice. Reprinted with permission from reference [88], 2023, American Chemical Society.

Li et al. [88] synthesized a deep eutectic solvent-based UV-curable resin with both hydrogen bonds and ionic interaction by mixing choline chloride (ChCl), acrylamide (AAm), and 4-Acryloylmorpholine (AcMo) (Figure 8a). The resin has an ultra-low viscosity (<0.1 Pa·s) and ultra-high curing rate, which is compatible with commercial LCD printers. The printed glassy components have high precision (50 microns) and optical transparency. The synergistic hydrogen bonds between hydroxyl, amino, and carbonyl groups in the polymer chains provide self-healing properties (75.9%). This polymer shows an extremely high stiffness and humidity-related conductivity, rendering it suitable for manufacturing humidity-responsive devices. In addition, two-dimensional structural assemblies with different sizes and their solubility in recycling and re-modeling were demonstrated in Figure 8b–d. The presence of choline chloride contributes to the solubility of the printed products, enabling sacrificial mold manufacturing. It is convenient for the manufacturing of precise, multi-functional structures on demand.

Zhu et al. [89] reported a dynamic polymer with highly customizable mechanical performance. Owing to the ionic and hydrogen bonds inside the polymer, the DLP-printed samples have good self-healing and recycling abilities. In addition, the printed products were easily tuned from soft elastomers to rigid plastics by adjusting the ratio of urethane monoacrylate/acrylic acid monomers. The on-demand preparation of complex structures, various assembly categories, and healable functional devices was realized by using this polymer. To a certain extent, this approach solves the environmental problems caused by traditional DLP thermosetting products. Soft and rigid models that were printed showed a good recovery effect in shape and strength after healing at 90 °C for 12 h. The printed products were recovered via thermocompression to obtain uniform samples. The tensile strength recovery rate, fracture strain recovery rate, and Young’s modulus recovery rate were determined to be 92%, 85%, and 109%, respectively. After recycling them multiple times, a 70% restoration of the mechanical performances was still observed.

Wu et al. [90] synthesized a solid conductive ionic gel (SCIg) with a rapid curing rate and self-healing function. A precursor solution composed of acrylic acid and choline chloride was mixed with a gelatin cross-linking agent containing many amino acid residues in the molecular chain. The acrylic acid and choline chloride formed a polymer network with inherent conductivity and the remaining amino acids generated a second rigid network, which allowed for the preparation of a new type of SCIg with excellent comprehensive performance. The rich hydrogen bonding sites in the system enhance the non-covalent interaction and afford excellent mechanical performances. Moreover, the addition of glycerol enhances the physical cross-linking networks and improves the healing ability (>95%). Based on the conductivity of the polymer, a stretchable resistive sensor, a compressible capacitive sensor, and an electronic skin were constructed by DLP, demonstrating the application of SCIg in flexible wearable devices. This SCIg achieves a balance between ionic conductivity and mechanical performance. Problems such as solvent leakage and the evaporation of gel elastomers containing liquid can be avoided by using SCIg. In addition, its rapid curing rate and high printing accuracy make it highly promising for application in the fields of electronic skin, physiological signal detection, and human–machine interfaces.

Invernizzi [91] and Suriano [92] et al. developed an SMP that was obtained via the crosslinking reaction of diol-terminated polycaprolactone (PCL) with 2-isocyanate ethyl methacrylate and methacrylate monomers. The PCL segment provides a shape memory function, and the quadruple hydrogen bond established by ureidopyrimidinone provided self-healing performance. Experiments proved that the DLP-printed ‘L’-type specimen shows similar tensile strength to the cast specimen The ureidopyrimidinone moiety does not harm the mechanical performances. The healed samples are still suitable for applications such as soft actuators.

Durand-Silva et al. [93] synthesized methacrylate monomers with aliphatic or aromatic urea motifs, which can exert a good dilution effect on oligomers. The presence of different types of hydrogen bonds improved the toughness and self-healing ability of the products. The cross-linking density increased with the increase in the urea group content. By controlling the content of hydrogen bonds, the mechanical strength was increased by 119% and the toughness was increased by 205%. They studied the effect of pendant aliphatic and aromatic ureas on the mechanical performances of polymer networks. As described in previous reports [94], they found that the presence of pendant hydrogen-bonded urea enhanced the mechanical tensile strength and toughness without reducing the elongation.

In the hydrogel system that is rich in hydrogen bonds, the mechanical strength and self-healing ability of the polymer can be further enhanced by introducing ion interactions, and the resulting conductivity can broaden the application scope of the polymer.

Huang et al. [32] developed a thermoplastic polymer composite with self-healing (>60%), efficient recyclability, and a customizable mechanical performance for DLP. The polymer contains soft and hard regions. The introduction of zinc methacrylate provides the composite with a second dynamic bonding (ionic bonding). The soft mono-functional urethane chains and ionic bonds suppresses the brittleness by acting as energy absorption units between the hard 4-Acryloylmorpholine segments. The complementarity of hard and soft chain segments considerably improves the mechanical strength of the component and the self-healing performance, thus facilitating the combination of the components with different mechanical properties. This is important for the re-assembly or recycling of robotic components after failure. Stemming from the superior combination of self-healing polymers, it outperforms most reported self-healing polymers, with a tensile strength and elastic modulus of up to 49 and 810 MPa, respectively. This study introduces self-healing and printing functions into a polymer without affecting its recyclability and strength, equilibrium self-healing ability, or mechanical performance.

Wu et al. [95] proposed an inter-penetrating network hydrogel based on poly(acrylic acid–N-vinyl-2-pyrrolidone) and carboxymethyl cellulose. They were physically cross-linked through Zn^2+^ coordination and hydrogen bonding. The printed hydrogel exhibits the same dynamic dual-physical interactions as ‘sacrificial bonds’ [96], resulting in a high tensile toughness (3.38 MJ·m^−3^) and good self-healing ability (*η*_stress_, 81%; *η*_strain_, 91%). The hydrogel was subjected to 40%-strain tensile cycles 100 times, and resistance responsiveness was still observed. The hydrogel can also be customized for flexible sensor printing. Owing to the designability and high resolution of its 3D printing (the profile is still smooth and complete at a minimum scale of 100 μm), the hydrogel has broad application prospects and avoids the limitations of the hydrogel-extrusion-based printing technology. In terms of resolution and printing speed, this hydrogel is expected to prepare wearable, flexible sensors.

### 3.2. Crystallization

Crystallization [97,98] is a process in which atoms or molecules are arranged according to certain rules to form crystals. Generally, polymers do not form regular single crystals owing to their long chain structure and chain entanglement behavior. However, polymers with ordered structures can form crystal embryos, from which crystals can eventually grow as the ordered structure cools from the amorphous state. Above the melting temperature (*Tm*) of the crystal, the crystalline polymer flows. When the polymer is damaged, a segment can flow to the damaged place. Upon the temperature decreasing, the crystal is re-formed. Therefore, crystalline polymers can also be self-healing.

**Figure 9:**
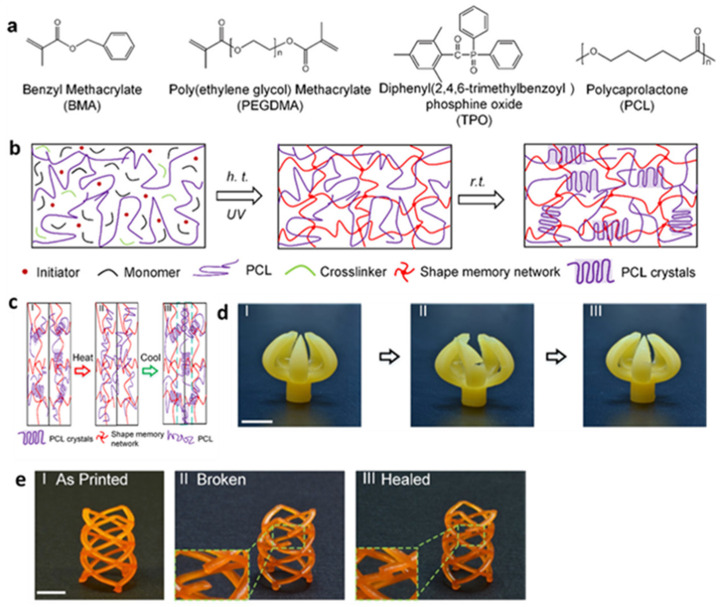
(**a**) Chemical structures of the components in SH-SMP solution. (**b**) Chemical structural evolution of the SH-SMP solution during UV-based 3D printing at high temperature (h.t.) and cooling down to room temperature (r.t.). (**c**) Illustrations of the self-healing mechanism. (**d**) 3D-printed gripper. (**I**) As-printed gripper. (**II**) Cut gripper. (**III**) Healed gripper. (**e**) 3D-printed stent. (**I**) As-printed. (**II**) Broken. (**III**) Healed (scale bars: 4 mm). Reprinted with permission from reference [99]. 2019, American Chemical Society.

Zhang et al. [99] incorporated a semi-crystalline linear PCL (relatively low melting temperature of 60 °C) with good miscibility on a system of SMP methacrylate (Figure 9a). After heating the prepared spline above 60 °C, the PCL crystal domain melts and the PCL linear chain is diffused through the boundary between two separated samples. When cooled to room temperature, the PCL linear chains formed crystalline domains. Some crystal domains cross the boundary between individual strips and combine them to achieve a repair effect (Figure 9b,c), which constitutes a solution to the irreparable thermosetting polymers. The prepared dual-network SMP exhibits a UV-curable self-healing ability (self-healing shape memory polymers, SH-SMP) and good compatibility with DLP 3D printing. This polymer is suitable for manufacturing complex printing structures with high resolution (up to 30 μm) (Figure 9d,e). The advantages of the system were demonstrated by printing some samples, including an SH-SMP holder and a cardiovascular stent. The potential application prospects of this system in the fields of soft robots, flexible electronics, and biomedical equipment were proven.

Wen et al. [100] mixed PCL with a UV-curable monomer to print a semi-interpenetrating polymer network elastomer with shape memory, self-healing function, and high tensile strength (20 MPa). The appearance of the sample after cutting and repairing was the same as the original, and the shape recovery speed decreased only slightly.

Abdullah et al. [101] proposed a temperature-responsive hydrogel. The hydrogel consists of hydrophilic poly (acrylic acid) chains containing different molar fractions of the hydrophobic segment C16A. The physical cross-linking of co-polymer chains can be achieved via hydrophobic association between the crystalline domains of hydrophobic segments. The melting and crystallization temperatures of the hydrogels are 38–40 °C and 25–29 °C, respectively. It allows for inducing a shape-memory function at temperatures close to the human body by optimizing the C16A content. By adjusting the molar fraction of the hydrophobic segment, the printed hydrogel can be made to exhibit a Young’s modulus of 23–215 MPa and toughness up to 7 MJ·m^−3^, enabling a transition from brittleness to toughness. The developed 4D-printable hydrogel has great potential in various biomedical applications.

### 3.3. Host-Guest Interaction

Host–guest interactions [102] are mainly caused by hydrophobic interactions and complementary shape and size characteristics between the host and guest molecules. The dynamic interaction between the host and guest molecules can endow the polymer with a self-healing function.

Wang et al. [34] prepared a three-arm chain segment based on an effective host–guest inclusion interaction between ethyl-acrylate-modified β-cyclodextrin (β-CD-AOI2) and acryloyl-tetraethylene-glycol-modified adamantane (A-TEG-Ad). A host–guest supramolecular hydrogel (HGGelMA) was obtained by the reaction of double bonds at the end of the arms. The biocompatible natural matrix gelatin methacryloyl (GelMA) formed a covalent cross-linking network. The resulting hydrogel has non-covalent bonds embedded in the covalent connection network. Weak non-covalent bonds can be quickly re-established through host–guest recognition after cleavage, while strong covalent bonds maintain the network. The hydrogel has excellent biodegradability and low immune rejection of natural hydrogels as well as a high strength, anti-fatigue performance, and rapid healing rate. The shear-thinning behavior and suitable viscosity of the HGGelMA precursor meet the requirements for 3D printing. The hydrogel is a promising printing biomaterial with potential biomedical applications, circumventing some of the issues of traditional hydrogels.

The reversibility and repair processes of reversible covalent bonds require external energy stimulation, such as heating, light, and catalysts. For example, the temperature required for the self-healing of transesterification bonds and Diels–Alder bonds is usually higher than 100 °C, and the stimulation does not promote efficient healing in common environments. Although reversible non-covalent bonds can often achieve satisfactory healing effects under mild conditions, the mechanical performances are usually not as good as those formed by dynamic covalent interactions. Moreover, hydrogen bonds are usually repaired at room temperature, but their mechanical strength is limited. Both types of bonds have advantages and disadvantages.

## 4. Challenges and Prospects

Dynamic interactions endow UV-curable 3D-printed polymers with a self-healing function and recyclability, prolong the service life of products, and alleviate environmental pollution and resource shortages. The research field of dynamic UV-curable polymer 3D printing is gradually developing, and the accompanying difficulties and challenges must be solved urgently. Future research should address the following issues:

(1) Photocurable 3D printing technology requires photosensitive resin with a low viscosity, but the molecular weight of low-viscosity resin is small, which will make the cross-linking density of the cured material high, causing the material to become hard and brittle. If the molecular weight of the resin is large, a large amount of monomer dilution is required, which will cause the resin to lose its original performance. The contradiction between resin viscosity and performance needs to be solved urgently;
(2) Balancing the mechanical performance and self-healing function of UV-curable self-healing polymers is the main goal. To achieve high mechanical performance, dynamic bonds with high bond energy are required, which decreases the self-healing efficiency of the polymer. At present, dual dynamic network structures [103,104,105] and multi-phase design [106,107] have been introduced into polymers and have achieved certain results, but new methods still need to be explored;
(3) UV-curable, self-healing polymers for 3D printing require external stimuli to activate damage healing; however, the stimulation intensity required for healing cannot be easily provided in practical applications. Developing polymers that can self-heal at room temperature or lower is more valuable for practical applications;
(4) Currently, the preparation of self-healing photosensitive resins is generally complicated and requires cumbersome steps. Therefore, simplification of the synthesis process, improvement of the yield, and the reduction of waste are necessary;
(5) Photocuring printing equipment is usually expensive and mainly used for printing small devices. Using dynamic interaction, it is undoubtedly convenient to construct large-size devices through module assembly.

## 5. Conclusions

The demand for practical applications has prompted the rapid development of UV-curing 3D printing technology. However, it is necessary to simultaneously introduce self-healing and other functions into the printing polymers to develop functional photosensitive resins. This paper describes photosensitive resins with dynamic bonding for 3D printing that have been studied in recent years and categorizes the types of dynamic bonding. The design, preparation, and application prospects of polymers are reviewed. The advantages and disadvantages of the printed products are summarized. It is hoped that this review will be of some help for the subsequent development of UV-curing, self-healing polymers with excellent properties.

## Data Availability

No new data were created.
